# Comparative sequencing and SNP marker validation for oat stem rust resistance gene *Pg6* in a diverse collection of *Avena* accessions

**DOI:** 10.1007/s00122-022-04032-z

**Published:** 2022-02-03

**Authors:** Tyler C. Gordon, Yue Jin, Nicholas A. Tinker, Wubishet A. Bekele, Samuel Gale, Harold Bockelman, J. Michael Bonman

**Affiliations:** 1 Small Grains and Potato Germplasm Research Unit, USDA-ARS, 1691 South 2700 West, Aberdeen, ID 83210 USA; 2 Cereal Disease Laboratory, USDA-ARS, 1551 Lindig Street, St. Paul, MN 55108 USA; 3 Ottawa Research and Development Centre, Agriculture and Agri-Food Canada, 960 Carling Ave, Ottawa, ON K1A 0C6 Canada

## Abstract

**Key message:**

Comparative sequence analysis was used to design a SNP marker that aided in the identification of new sources of oat stem rust resistance.

**Abstract:**

New races of *Puccinia graminis* f. sp. *avenae* (*Pga*) threaten global oat production. An *A. strigosa* accession known to carry the broadly effective oat stem rust resistance gene, *Pg6*, was crossed with two susceptible *A. strigosa* accessions to generate 198 F_2:3_ families and 190 F_5:6_ RILs. The RIL population was used to determine that *Pg6* was a single dominant gene located between 475 and 491 Mbp on diploid chromosome AA2 of the *A. atlantica* genome. This region was further refined by identifying SNPs associated with *Pg6* resistance in a panel of previously sequenced A-genome accessions. Twenty-four markers were developed from SNPs that showed perfect association between the *Pg6* phenotype and 11 sequenced *Avena* diploid accessions. These markers were validated in the RILs and F_2:3_ families, and the markers most closely linked with resistance were tested in a diverse panel of 253 accessions consisting of oat stem rust differentials, all available diploid *Avena* spp. accessions, and 41 *A. vaviloviana* accessions from the National Small Grains Collection. One SNP marker located at 483, 439, 497 bp on AA2, designated as AA2_483439497, was perfectly associated with the *Pg6* phenotype in *Avena strigosa* diploids and was within several Kb of a resistance gene analog, RPP13. The marker results and seedling testing against *Pga* races DBD, KBD, TJS, and TQL enabled the postulation of *Pg6* and potential new sources of resistance in the *Avena* panel. These results will be used to infer *Pg6* presence in other germplasm collections and breeding programs and can assist with introgression, gene pyramiding, and cloning of *Pg6*.

**Supplementary Information:**

The online version contains supplementary material available at 10.1007/s00122-022-04032-z.

## Introduction

Oat (*Avena sativa* L.) is an important cereal crop with 23 million t of oat grain harvested globally in 2018 (FAOSTAT 2020). Dehulled oat groats are high in protein, antioxidant polyphenols, and saponins. Oat grain is high in *β*-glucan soluble fiber, which can lower plasma glucose and cholesterol levels (Pomeranz et al. 1971; Fardet 2010). Oat is also used extensively as grain and forage for cattle, horses, and poultry and can provide a nutritive complement or organic alternative to other animal feed (Federizzi and Mundstock 2004; Winkler et al. 2018).

Oat stem rust caused by the fungal pathogen *Puccinia graminis* f. sp. *avenae* Erikss. and Henning (*Pga*) is an economically important foliar disease of oat. Urediniospores of *Pga* rapidly proliferate on susceptible oat cultivars and can cause severe yield and quality losses under favorable environmental conditions (Roelfs and Long 1980; Martens 1985). Van Niekerk et al. (2001) demonstrated that after an experimental stem rust epidemic, oat grain yield and test weight were reduced by 85% and 45%, respectively. Historically, oat profit margins were low (Hoffman and Livezey 1987) making genetic resistance an attractive, cost-efficient form of disease control for this crop.

Stakman et al. (1923) first documented physiologic races of *Pga* and specific virulence patterns on a set of three differential oat varieties. Since then, 15 unique, numbered oat stem rust resistance (*Pg*) genes and the *Pg-a* complex have been described (Fetch and Jin 2007). Many of the *Pg* resistance genes are ineffective at high temperatures and were originally described in hexaploid oat accessions (Fetch and Jin 2007; Boshoff et al. 2019). Resistance conferred by *Pg6*, originally identified in the A-genome diploid, *Avena strigosa* Schreb. accession CIav 6956, is temperature insensitive and has been widely effective against North American *Pga* isolates. Of the 77 North American oat stem rust races evaluated by Fetch and Jin (2007), only two, NA1 and NA70, showed virulence to *Pg6*. Similarly, a survey of oat stem rust isolates from a recent epidemic in Hebei Province, China, detected virulence to all *Pg* genes except *Pg6* and *Pg15* (Li et al. 2015). However, virulence surveys of oat stem rust isolates from oat-producing regions in Australia and South Africa have detected a high frequency of isolates with virulence to *Pg6* (Adhikari et al. 2000; Boshoff et al. 2019). Steinberg et al. (2005) evaluated 9978 *Avena* spp. accessions from the Canadian, US, and Israeli national germplasm repositories and concluded that *A. strigosa* accessions might harbor novel sources of resistance. Unfortunately, field-resistant accessions from their study (Steinberg et al. 2005) were susceptible to oat stem rust race NA1, which indicated the widespread presence of *Pg6* in the tested group of accessions.

Race-specific oat crown rust resistance has been quickly overcome in North America due to rapidly evolving *Puccinia coronata* Corda. f. sp. *avenae* Eriks. (*Pca*) populations on the alternate host, buckthorn, *Rhamnus cathartica* L. (Carson 2008). In contrast, the *Pga* population may be more stable in North America due to eradication efforts of the stem rust alternate host, barberry (*Berberis vulgaris* L.). For a closely related disease, wheat stem rust, combining two to three adult plant resistance (APR) genes in a single cultivar, has been shown to be an effective control strategy (Rouse et al. 2014; Kosgey et al. 2021). There are two known APR oat stem rust sources, *Pg11* and *Pg17* (Fetch and Jin 2007), but *Pg17* has been reported to have extremely high levels (70 to 90% severity) of disease in oat stem rust field trials in Canada (Steinberg et al. 2005). To protect oat production from oat stem rust epidemics, it is imperative that oat rust surveys continue to document pathogen race diversity and new sources of effective resistance identified and characterized.

Molecular markers closely linked with known stem rust resistance genes provide a quick way to identify germplasm with potentially novel resistance. RFLP markers have been developed for *Pg9* near an oat prolamin gene, pOp6 (O’Donoughue et al. 1996). SNP markers have recently been developed for *Pg2* at 241 cM on Mrg 20 (Kebede et al. 2020b) and *Pg13* between 67 and 69 cM on Mrg 18 (Kebede et al. 2020a). Recently, the first genome sequences for the A-genome *A. atlantica* and C-genome, *A. eriantha* (Maughan et al. 2019), and a publically released genome reference for the ACD-genome, *A. sativa* (PepsiCo 2020), became available. These pseudo-molecule, reference-quality sequences may provide access to additional molecular variants with close linkage to resistance loci.

The purpose of this study was to design diagnostic molecular markers for *Pg6* in order to determine if diploid *Avena* accessions offer new sources of stem rust resistance. This goal was accomplished by (1) genetic mapping of the *Pg6* locus in two bi-parental mapping populations, (2) comparative sequence-based SNP marker development within the *Pg6* target region in *A. strigosa* accessions, (3) screening of available diploid oat accessions from the National Small Grains Collection (NSGC) with *Pg6*-specific *Pga* races and markers, and (4) identifying accessions with unique resistance for further study.

## Materials and methods

### Population development

An *A. strigosa* accession carrying *Pg6*, CIav 6956, was crossed as the pollen parent with two *A. strigosa* accessions susceptible to stem rust, CIav 2524 and PI 573582. The resulting populations were denoted as 2524/*Pg6* and 573582/*Pg6*. The 2524/*Pg6* population consisted of 198 F_2:3_ families, and the 573582/*Pg6* consisted of 190 F_5:6_ recombinant inbred lines (RIL) generated by single seed descent.

### *Avena* spp. validation panel

Accessions of all of the diploid *Avena* spp. available from the NSGC were selected for *Pg6* validation including: *A. atlantica*, *A. brevis*, *A. nuda.*, *A. strigosa*, *A. wiestii*, *A. damascena*, *A. longiglumis*, *A. eriantha*, and *A. ventricosa* (Table 1). Four of these species including *A. atlantica*, *A. brevis*, *A. strigose,* and *A. wiestii* were previously shown to compose a single A-genome species complex. An additional 41 accessions of the tetraploid AB-genome oat, *A. vaviloviana* (Malzev) Mordv., were selected based on reports of novel intermediate levels of field resistance in this species and its previous diploid classification (Steinberg et al. 2005). Together, there were 253 accessions selected for *Pg6* resistance validation including 198 diploid accessions, 41 tetraploid accessions, 12 *A. sativa* stem rust differentials obtained from the USDA-ARS Cereal Disease Lab (CDL), and three susceptible *A. sativa* cultivars ‘Marvellous,’ ‘Otana,’ and ‘Rodney 0’ (Supplementary Table S1).

**Table 1:** *Avena* species genome assignment, ploidy, number of accessions tested, and *Pg6* postulation

Avena species	Genome^a^	Ploidy	No. tested	*Pg6* phenotype^b^	*Pg6* genotype^c^	Other resistance^d^
*A. atlantica* B. R. Baum and Fedak	A_s_	2*n*	2	0	0	2
*A. brevis* Roth	A_s_	2*n*	22	2	2	0
*A. damascena* Rajh. & B. R. Baum	A_d_	2*n*	3	0	0	0
*A. eriantha* Durieu	C_p_	2*n*	9	0	0	0
*A. longiglumis* Durieu	A_l_	2*n*	17	6	1	2
*A. nuda* L	A_s_	2*n*	9	0	0	0
*A. strigosa* Schreb	A_s_	2*n*	127	46	41	5
*A. ventricosa* Balansa ex Coss	C_v_	2*n*	2	0	1	0
*A. wiestii* Steud	A_s_	2*n*	6	2	1	0
*A. vaviloviana* (Malzev) Mordv	AB	4*n*	41	0	0	11
*A. sativa* L	ACD	6*n*	15	0	0	13

^a^Genome assignment based onYan et al. (2016)

^b^Based on a typical *Pg6* phenotypic response of 0; or;13 to races DBD, KBD and TJS and an IT of 3 or 4 to TQL

^c^Number of accessions that carry the *Pg6* resistant-associated allele for AA2_483439497

^d^Accessions with resistance reactions that are not typical of *Pg6*, as described above

### Inoculation and phenotyping

Seedling phenotyping was carried out as previously described for the CDC Boyer/GS-7 population in Kebebe et al. (2020a). Oat stem rust race KBD (virulence pattern shown in Table 2) was used to inoculate seedlings of the 573582/*Pg6* and 2524/*Pg6* populations in three and four independent replications, respectively. Within each test, two seeds per family were planted into containers (3.8 mm diameter × 210 mm depth, Stuewe & Sons, Inc., Tangent, OR). Three replicates of each parent were included in each population, and the planting order was randomized within each replication. Oat stem rust races DBD, KBD, TQL and TJS (Table 2) were used in separate tests to inoculate the 253 *Avena* accessions within the diversity panel and postulate the presence of *Pg6*. Race TJS is virulent to all known stem rust resistant genes except *Pg6*, *Pg10*, and *Pg16* while TQL is virulent to *Pg6* and was used to postulate *Pg6* presence. Seedling infection types (IT) were recorded on the first seedling leaves 14 days after inoculation based on the 0 = fully resistant to 4 = fully susceptible scale developed by Stakman et al. (1962). Seedlings were classified as resistant if they had an IT below 3 and susceptible if they had an IT of 3 or above. Individual accession phenotype data for each of the four oat stem rust races are listed in Supplementary Table S1 and are available from the US National Germplasm System online database: Germplasm Resources Information Network (GRIN), accessed at https://npgsweb.ars-grin.gov/gringlobal/search.aspx.

**Table 2:** *Puccinia graminis* f. sp. *avenae* races used and number of accessions resistant to each race

Race^a^	Isolate	Effective/ineffective *Pg* genes	Number of accessions^b^	
			Resistant	Susceptible
DBD	05ID107	***1, 2, 4, 6, 8, 9, 10, 12, 13, 16/*** *3, 15*	87	147
KBD	14ID001	***1, 6, 8, 9, 10, 12, 13, 16/*** *2, 3, 4, 15*	71	166
TJS	07ND124	***6, 10, 16/*** *1, 2, 3, 4, 8, 9, 12, 13, 15*	64	166
TQL	11TX004-8	***9, 10, 13, 15, 16/*** *1, 2, 3, 4, 6, 8, 12*	17	219

^a^Based on the letter code system of nomenclature for *Pga* (Fetch and Jin 2007)

^b^Accessions tested from the 253 *Avena* spp. diversity panel

Pg genes shown in bold are effective to the the race in the corresponding row

### Genetic mapping

DNA was extracted from leaf tissue following the protocol described by Sika et al. (2015). A 2 cm section of leaf tissue from seedlings with two or three leaves was collected into 96-well Corning® Costar® tubes (Corning, NY). Tissue was macerated in an extraction buffer composed of 1% sodium dodecyl sulfate, and 5 M NaCl, and the resulting homogenate was spun at 3500 rpm for 15 min. Supernatant was washed with 500 µl of 2-propanol, placed on ice for 5 min, then spun at 3500 rpm for 15 min. Resulting DNA pellets were washed with 500 µl of 70% ethanol and suspended in 100 µl of 10 mM Tris–HCl pH 8.0.

DNA samples from 140 F_5:6_ families and two parent replicates from the 573,582/*Pg6* population were sent to the USDA-ARS Small Grains Genotyping Laboratory in Fargo, ND, where they were genotyped using the 6 K iSelect SNP assay as described by the manufacturer (Illumina, San Diego, CA). Manual allele clustering was performed using Genome Studio v.2.0.2 (Illumina). JMP Genomics v.9.0 (SAS Institute Inc., Cary, NC) was used to filter curated markers for all subsequent linkage analyses. Heterozygous calls were excluded from the analysis as were markers with minor allele frequency (MAF) < 5%, or missing data > 10%. Within this *A. strigosa* population, 4514 of the 4852 Illumina SNP markers either did not amplify or were monomorphic between the parents. A final set of 238 markers and 136 families and the parents were used to perform linkage analysis.

Stem rust infection types were coded so that 0 = susceptible, 1 = mixed or segregating, and 2 = resistant for preliminary linkage analysis. Using JMP Genomics v.9.0, SNP markers were assigned to seven linkage groups, that expected for A-genome diploids, and genetic distances were calculated through the interactive hierarchical clustering and linkage map ordering processes using the Kosambi mapping function. Composite interval mapping was used to find LOD scores, additive effects, and percent variation explained by each marker.

### Bioinformatics and marker design

The recently published *A. atlantica* (diploid A-genome) reference genome sequence (Maughan et al. 2019) was used to locate the mapped genetic region by searching for SNP sequences using the Comparative Genomics (CoGe) BLAST function https://genomevolution.org/coge/CoGeBlast.pl. Ten accessions of *Avena strigosa* and its homotype *Avena brevis* that were previously sequenced by Maughan et al. (2019) were identified for which consistent disease phenotypes were available. An additional sequenced accession with unknown phenotype (PI 436102) was included for diagnostic purposes. These 11 previously sequenced accessions (Supplementary Table S2) were analyzed for additional resistant associated SNPs within the genomic region linked with *Pg6* resistance between 475 and 490 Mbp using Fast-WGS (Torkamaneh et al. 2018). Fast-WGS employed BWA version 0.7.17 to map paired-end Illumina sequence reads of whole-genome shotgun libraries with a minimum base quality of 20 to the *A. atlantica* reference genome. Mapped reads with minimum map quality of 20 were sorted by Samtools version 0.1.19 to generate sorted BAM files. Platypus version 0.8.1.1 (Rimmer et al. 2014) was used to call sequence variants with a minimum of two reads per variant and create the output VCF file. The VCF file was filtered with VCFtools version 0.1.16 (Danecek et al. 2011) to retain only bi-allelic SNPs in the identified map region.

Results in VCF format were further filtered to specifically identify SNP variants that were diagnostic between resistant and susceptible accessions and then to record these as simple genotype strings, where ‘0’ represents the reference allele (*A. atlantica*), ‘1’ represents the alternate allele, ‘h’ is a heterozygote, and ‘x’ is missing data. SNPs that matched string patterns corresponding to perfect associations (i.e., the string patterns described in Supplementary Tables S2 and S3) were recorded for further work, while a set of 13 SNPs that did not match these strings were selected randomly as non-associated controls. An in-house script was used to extract 50 bp of context sequence on either side of each target SNP from the *A. atlantica* reference genome and to produce a SNP design string showing all target and non-target SNPs within this design string (Supplementary Tables S4 and S5).

Competitive SNP molecular marker assays were designed by 3CR Bioscience (Harlow, UK). SNP assays were designed from the two Illumina SNP sequences that flanked either side of the *Pg6* locus in the RIL population, and 24 assays were designed from the *A. atlantica* sequence design strings. Only sequences with the primary target SNP and $\le$ 1 additional SNP were used for primer design (Supplementary Table S6). Primers were ordered through IDT (Coralville, IA). Assays were tested according to the PACE™ master mix protocol described by 3CR Bioscience (Harlow, UK). In each well, 5 µl of gDNA (20 ng/µl), 0.138 µl of assay mix (12 µM of each competing forward primer, 30 µM of the common primer and 46 µM of water), and 5 µl of 2X PACE master mix were added. Cycling conditions were 94 °C for 15 min, 10 cycles of 94 °C for 20 s and 65 °C for 60 s with an annealing temperature decrement of 0.8 °C per cycle, and 30 cycles of 94 °C for 20 s and 57 °C for 60 s. When cycling had ended, assays were read for FAM and HEX fluorescence with a CFX96 or CFX384 (BioRad, Hercules, CA). Assays that showed SNP polymorphism between accession CIav 6956 (*Pg6* carrier) and the susceptible parents were tested in the 573582/*Pg6* RIL population. A subset of markers that were closest to the resistance locus were validated in the 2524/*Pg6* population and the diverse panel of 253 *Avena* spp. accessions described above.

## Results

### Seedling resistance

**Fig. 1:**
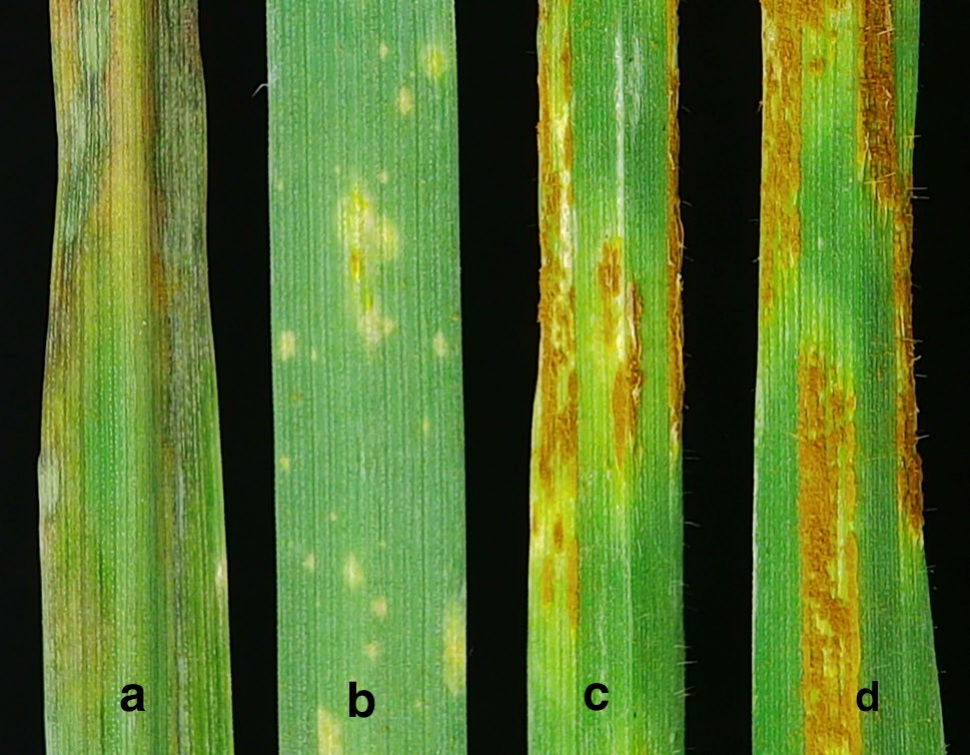
*Avena strigosa* primary leaf infection type (IT) phenotypes inoculated with oat stem rust race KBD and shown 14 dpi; from left to right, CIav 6956 carrying *Pg6* with two different IT **a** ; N and **b** ; 1, **c** Susceptible parent, CIav 2524, with IT 3 + and **d** susceptible parent, PI 573,582, with IT 4

When inoculated with *Pga* races DBD, KBD, and TJS, the *Pg6* differential accession CIav 6956 showed resistant ITs that ranged between ‘0;’ and ‘;13− ’ and the susceptible parents, CIav 2524 and PI 573,582, had ITs that ranged between ‘3’ and ‘4’ (Fig. 1, Supplementary Table S1). The 2524/*Pg6* F_2:3_ population fit a single dominant gene model segregation ratio with 88 resistant families, 61 segregating families and 49 susceptible families across four trials (*χ*^2^ = 3.9, *P* = 0.14). The 573582/*Pg6* *F*_5:6_ population RILs also fit a single gene model with 95 resistant lines, 6 segregating lines and 89 susceptible lines recorded across three trials (*χ*^2^ = 0.38, *P* = 0.83).

Seedling resistance to *Pga* races DBD, KBD, and TJS was widespread within the diversity panel. Approximately 30% of the *Avena* spp. accessions were resistant to these races (Table 1), but only 8 of the accessions showed resistance to race TQL. Accessions susceptible to *Pga* race TQL and with clear resistance ITs of 0; to;13 to *Pga* races DBD, KBD, and TJS were postulated to carry *Pg6.* Within the diversity panel, 56 such accessions were identified, and all were A-genome diploids, primarily from *A. strigosa* (Table 3).

**Table 3:** Accessions postulated to carry oat stem rust resistance gene, *Pg6* based on genotype (Based on SNP AA2_483439497) and infection type (Infection type scale according to Stakman et al. (1962) where 0 = immune and 4 = fully susceptible) when challenged with four *Pga* races

Accession	Species	Origin	Pg gene postulation	*Pg6* genotype	*Pga* race			
					DBD	KBD	TJS	TQL
PI 119009	*A. brevis*	Brazil	*Pg6*	+	;	;	;	3 +
PI 158204	*A. brevis*	Portugal	*Pg6*	+	1;	;4	;3 −	4
CIav 9088	*A. longiglumis*	Morocco	*Pg6*	–	;1	;1	;1	3 +
PI 657295	*A. longiglumis*	Morocco	*Pg6*	–	;1	;13	;1 +	4
PI 657342	*A. longiglumis*	Morocco	*Pg6-*mixed		;1	;1	;/3	4
PI 657386	*A. longiglumis*	Morocco	*Pg6-*mixed	–	;1	;1	;/3 −	3
PI 657388	*A. longiglumis*	Morocco	*Pg6*		;		;3 −	3
PI 657389	*A. longiglumis*	Morocco	*Pg6*		;		;1 −	3
CIav 2525	*A. strigosa*	UK	*Pg6*-mixed	+ /–	3 +	;/4	4	4
CIav 4639	*A. strigosa*	Brazil	*Pg6*	+	0;	;	0;1	4
CIav 5057	*A. strigosa*	Soviet Union	*Pg6*	+	;N3 −	;N	;N3 −	4
CIav 5082	*A. strigosa*	Uruguay	*Pg6*	+	0;	;	0;	4
CIav 6858	*A. strigosa*	Uruguay	*Pg6*	+	0	;	;13 −	3 +
CIav 6956	*A. strigosa*	Canada	*Pg6* differential	+	;1-	1	;1	4
CIav 7010	*A. strigosa*	Brazil	*Pg6*	+	0;	;	0;	3 +
CIav 7280	*A. strigosa*	USA	*Pg6*	+	0	;	0;1 −	3 +
CIav 8087	*A. strigosa*	Spain	*Pg6*	+	;	;	;1	3 +
CIav 8089	*A. strigosa*	USA	*Pg6*	+	0	0;	0;	3 +
CIav 9020	*A. strigosa*	Argentina	*Pg6*	+	0;	;	0;	3 +
CIav 9021	*A. strigosa*	Canada	*Pg6*	+	0	;	0	3 +
CIav 9035	*A. strigosa*	Russia	*Pg6*	+	;	;	;	3
CIav 9038	*A. strigosa*	UK	*Pg6*	+	;	;	;	3
CIav 9065	*A. strigosa*	Canada	*Pg6*	+	;	;	;	3 +
CIav 9066	*A. strigosa*	Canada	*Pg6*	+	;1	;	;/3 −	3
PI 158245	*A. strigosa*	Spain	*Pg6*	+	;	;1	;	3
PI 158246	*A. strigosa*	Spain	*Pg6*	+	0	;1	;	3
PI 186606	*A. strigosa*	Brazil	*Pg6*	+	0;	;	;	3
PI 244466	*A. strigosa*	Brazil	*Pg6*	+	0;	;	;1	3 +
PI 244470	*A. strigosa*	Brazil	*Pg6*	+	;	0	;1	3 +
PI 244471	*A. strigosa*	Brazil	*Pg6*-mixed	+ / −	;/3	;/4	;/3	3
PI 244472	*A. strigosa*	Brazil	*Pg6*	+	0;	;	;1 −	3
PI 258730	*A. strigosa*	Spain	*Pg6*	+	0;	0;	;	3
PI 258731	*A. strigosa*	Spain	*Pg6*	+	;1	;3 N	;1	3 +
PI 258733	*A. strigosa*	Spain	*Pg6*	+	0;	;1-	;1	3
PI 291990	*A. strigosa*	Israel	*Pg6*	+	;	0;	;	4
PI 291991	*A. strigosa*	Israel	*Pg6*	+	0;	;	0;	4
PI 292226	*A. strigosa*	Israel	*Pg6*	+	0;	;	0;	4
PI 304557	*A. strigosa*	UK	*Pg6*	+	;	;1	;1	3
PI 436031	*A. strigosa*	Chile	*Pg6*	+	2;	;	0;N	4
PI 436080	*A. strigosa*	Chile	*Pg6*	+	0;	;1 N	;	4
PI 436081	*A. strigosa*	Chile	*Pg6-*mixed	–	;N	;/4	;N/3	4
PI 436103	*A. strigosa*	Chile	*Pg6*	+	0;	0;	0;	4
PI 436104	*A. strigosa*	Chile	*Pg6*	+	0;	0;	0	4
PI 436105	*A. strigosa*	Chile	*Pg6*	+	0;	;	0	4
PI 436106	*A. strigosa*	Chile	*Pg6*	+	0	0;	0	4
PI 436108	*A. strigosa*	Chile	*Pg6*	+	;N	;	;N/3	4
PI 436109	*A. strigosa*	Chile	*Pg6*	+	0	;	0	3 +
PI 436110	*A. strigosa*	Chile	*Pg6*	+	0;	;	0	4
PI 436111	*A. strigosa*	Chile	*Pg6*	+	0;	;1 N	0;	4
PI 436114	*A. strigosa*	Chile	*Pg6*	+	0;	0	0	4
PI 436117	*A. strigosa*	Chile	*Pg6*	+	0;	;	0;	4
PI 436118	*A. strigosa*	Chile	*Pg6*-mixed	+ / −	;/4	;/4	;1/3	4
PI 573584	*A. strigosa*	Spain	*Pg6*	+	;	;3	;2	3
PI 573585	*A. strigosa*	Spain	*Pg6*-mixed	+	4	;/4	0/3 +	4
CIav 9053	*A. wiestii*	Canada	*Pg6*	+		;N	;13 −/3	3 +
PI 657352	*A. wiestii*	Morocco	*Pg6*	–	;	;	;1	4

Twenty accessions exhibiting resistance had ITs across *Pga* races that did not match the expected *Pg6* phenotypic profile (Table 4). Only eight of the potentially novel sources were resistant to race TQL, and the others had resistance that was unique in another way. For instance, eight of the accessions were resistant to *Pga* race DBD, but susceptible to all the other tested races. Of the unique resistant accessions, 11 were tetraploids from Ethiopia and three of those, PI 412764, PI 412765, and PI 412768, had consistent ‘2’ ITs across races. Within the diploid group, PI 131695, PI 158247, and PI 657297 had the lowest ITs to race TQL.

**Table 4:** *Avena* accessions with unique and potentially novel oat stem rust resistance based on genotype (Based on SNP AA2_483439497) and *Pga* race phenotypes (Infection type scale according to Stakman et al. (1962) where 0 = immune and 4 = fully susceptible)

Accession	Species	Origin	*Pg* gene postulation	*Pg6* genotype	*Pga* race			
					DBD	KBD	TJS	TQL
PI 657294	*A. atlantica*	Morocco	?	–	4	;	4	4
PI 657393	*A. atlantica*	Morocco	?	–	;1 +	4	3	3
PI 657297	*A. longiglumis*	Morocco	?-mixed		;/3 −	;1	;/3	23
PI 657387	*A. longiglumis*	Morocco	?	–	;1	4	3	3
PI 131695	*A. strigosa*	Poland	?	–	;	;4	3	;4
PI 131640	*A. strigosa*	Poland	?	–	;	4	3	3 +
PI 158247	*A. strigosa*	Portugal	?	+	2	4	2	23 −
PI 186614	*A. strigosa*	Brazil	?	–	4	;4	4	4
PI 361911	*A. strigosa*	Romania	?	–	2	4	3 +	3 +
PI 412726	*A. vaviloviana*	Ethiopia	?	–	1 + 3 −	3 +	3	3
PI 412742	*A. vaviloviana*	Ethiopia	?	–	1 + 3	3	13	3 +
PI 412748	*A. vaviloviana*	Ethiopia	?	–	13 −	3	3-	3
PI 412749	*A. vaviloviana*	Ethiopia	?	–	2	2	2	22 +
PI 412751	*A. vaviloviana*	Ethiopia	?	–	13 −	3	3	3 +
PI 412752	*A. vaviloviana*	Ethiopia	?	–	13 −	3	3	3 +
PI 412764	*A. vaviloviana*	Ethiopia	?	–	2	2 +	2	2
PI 412765	*A. vaviloviana*	Ethiopia	?	–	2	2	2	2 −
PI 412766	*A. vaviloviana*	Ethiopia	?	–	2	2 +	2	3
PI 412767	*A. vaviloviana*	Ethiopia	?	–	2	2	3	2 −
PI 412768	*A. vaviloviana*	Ethiopia	?	–	2	2	2	2 −

### Marker development and validation

A sparse genetic map with seven linkage groups was constructed from 238 polymorphic SNPs generated from the 573582/*Pg6* F_5:6_ bi-parental population. Thirteen of the SNP markers were linked within 10 cM of the *Pg6* locus, and one Illumina SNP, GMI_ES02_c32129_380, showed a high LOD and additive effect values (Table 5). The resistance locus was initially mapped to a region between 475 and 491 Mbp on chromosome AA2 (scaffold ScoFOjO_1702_2338) with the closest Illumina SNP marker at 490 Mbp on AA2. Of the 13 closely linked Illumina SNP markers, 12 were mapped to the *A. atlantica* reference genome and their linkage map order was generally consistent with their physical positions (Table 5).

**Table 5:** Mapping the *Pg6* locus using *Pga* KBD IT phenotypes, 238 SNP and 136 RILs from the 573582/*Pg6* RIL population marker

	cM^a^	Mbp^b^	LOD	Additive^c^	*R*^2^
GMI_ES_CC7849_819	48.22	469.0	3.0	0.51	0.10
GMI_DS_LB_10925	47.84	484.6	4.4	0.64	0.15
GMI_GBS_37983	47.84	472.0	4.4	0.64	0.15
GMI_DS_LB_7139	47.84	472.3	4.4	0.64	0.15
GMI_DS_LB_2908	47.84	472.2	4.4	0.64	0.15
GMI_DS_LB_5657	47.84		4.4	0.64	0.15
GMI_ES15_lrc19156_98	47.84	470.6	4.4	0.64	0.15
GMI_GBS_9578	45.79	474.9	5.6	0.87	0.18
*Pga*_KBD_locus	40.6				
GMI_ES02_c32129_380	35.22	491.1	60.7	1.83	0.89
GMI_ES22_c12033_457	35.22	490.2	20.9	1.37	0.53
GMI_ES15_c16513_175	34.46	491.8	3.3	0.63	0.11
GMI_ES01_c25986_126	32.51	493.6	3.3	0.56	0.11
GMI_GBS_53244	30.62	495.2	1.9	0.41	0.07

^a^Linkage groups (LG) cM positions calculated from the SNPs and phenotypes within the population

^b^Physical positions on chromosome AA2 of the *Avena atlantica* genome sequence using Comparative Genomics (CoGe) BLAST

^c^Additive effect where stem rust infection types were coded so that 0 = susceptible, 1 = mixed or segregating, and 2 = resistant

**Fig. 2:**
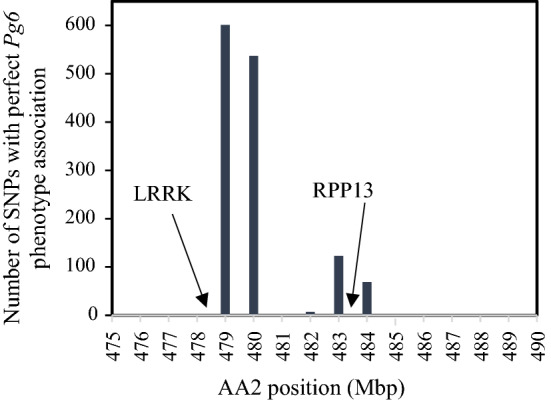
Number of SNP variants with perfect *Pg6* phenotype association across a group of 11 A-genome *Avena* accessions; candidate resistant genes are shown for reference

A total of 196,468 variant calls were made in the 15 Mbp sequenced target region associated with the *Pg6* locus across the 11 sequenced accessions shown in Supplementary Table S2. These variants were further filtered based on string patterns (Supplementary Table S3) to identify a set of 1,338 SNPs having one allele in all five putative *Pg6* accessions and the other allele in all five susceptible accessions (Supplementary Table S4). The sequence for this target region can be accessed in a genome browser hosted by GrainGenes (Blake et al. 2019) at the link provided in Supplementary Table S3. Interestingly, only strings 1 or 4 (Supplementary Table S3) were found, suggesting that the unknown accession PI 436102, formerly misclassified as *A. sativa*, is most likely an *A. strigosa* accession that caries the *Pg6* allele. All of the filtered *Pg6*-associated SNPs were located exclusively in three clusters between 478 and 484 Mbp, with the cluster between 478.4 and 479.4 Mbp showing the highest frequency of perfect associations (Fig. 2 and Supplementary Table S5).

SNP assays were designed for two of the flanking Illumina SNPs and 24 of the perfect *Pg6*-associated SNPs across the region of interest (Supplementary Table S6). Most of the assay designs were near two resistance gene analogs (RGAs). One RGA was a leucine-rich repeat receptor-like protein kinase (LRRK) in a 3-kb region beginning at 478,733,268 bp, while the other RGA was the 5 kb Resistance to Peronospora Parasitica protein 13 (RPP13) beginning at 483,423,997 bp. Thirteen of the assays were polymorphic between the *Pg6* carrier, CIav 6956, and the two susceptible *A. strigosa* parents, CIav 2524 and PI 573,582 (Table 6). SNPs near the LRRK RGA were between 3.4 and 5.3 cM and 6.4 and 10.1 cM relative to *Pg6* carrier status in the 573582/*Pg6* population and 2524/*Pg6* population, respectively. SNPs near the RPP13 RGA were perfectly aligned with the *Pg6* phenotypes in the 573582/*Pg6* population and between 0.0 and 4.9 cM relative to the *Pg6* carrier statusin the 2524/*Pg6* population.

**Table 6:** SNP marker analysis with genetic distances from the *Pg6* resistance locus in two bi-parental mapping populations and number of misclassified *Avena strigosa* accessions

Marker	Chr	Position (bp)	*R* SNP^a^	573,582/*Pg6* cM^b^	2524/*Pg6* cM^c^	Misclassified^d^
GMI_ES15_lrc19156_98	AA2	470,632,868	**C**/T	14.3	8.2	24
AA2_478733268	AA2	478,733,268	T/**C**	5.2	10.1	17
AA2_478733705	AA2	478,733,705	A/**C**	3.4	7.4	19
AA2_478736172	AA2	478,736,172	G/**A**	5.3	6.4	15
AA2_479335146	AA2	479,335,146	A/**C**	6.3		
AA2_479345016	AA2	479,345,016	**C**/T	11.0		
AA2_482018487	AA2	482,018,487	C/**T**	4.2		
AA2_482100965	AA2	482,100,965	C/**T**	2.6		
AA2_483427147	AA2	483,427,147	C/**G**	0.0	2.3	5
AA2_483429191	AA2	483,429,191	A/**G**	0.0	3.6	8
AA2_483439497	AA2	483,439,497	C/**T**	0.0	0.0	0
AA2_483451960	AA2	483,451,960	A/**G**	0.0	4.9	8
AA2_483503144	AA2	483,503,144	**C**/A	1.4		
AA2_485680524	AA2	485,680,524	C/**T**	5.3		
GMI_c32129_380	AA2	491,081,975	**C**/T	12.2	5.6	22

^a^SNP *underlined* and in bold type is associated with the resistant parent

^b^Centimorgans from the *Pg6* resistance locus in the 573582/*Pg6* F_5:6_ mapping population

^c^Centimorgans from the *Pg6* resistance locus in the 2524/*Pg6* F_2:3_ mapping population

^d^Number of *A. strigosa* accessions, of the 127 tested, that showed a misclassification between allelic call and phenotype

Selected SNP assays showing close linkage with the resistance locus in the mapping populations were well aligned with postulated *Pg6* phenotypes in the diverse panel of 253 *Avena* accessions (Supplementary Table S1 and Fig. S1). The SNP assay showing the best association with *Pg6* resistance, AA2_483439497, corresponded perfectly with the *Pg6* phenotype of every *A. strigosa* accession (Table 6, Supplementary Table S1). Additionally, AA2_483439497 correctly differentiated every *A. sativa Pg* differential, tetraploid *A. vaviloviana* accession, and showed failed reactions in all but two of the C-genome *A. eriantha* and *A. ventricosa* accessions (Supplementary Table S1).

## Discussion

In the present study, 56 *Avena* accessions were postulated to carry *Pg6* and 20 *Avena* accessions were identified with potentially novel resistance (Table 4). Unique resistance was rare in the diploids and only three accessions, PI 158247, an *A. strigosa* accession from Portugal, PI 131695 an *A. strigosa* accession from Poland and PI 657297 an *A. longiglumis* accession from Morocco showed moderate resistance to race TQL. Additionally, PI 186,614, from Rio Grande do Sul, Brazil, had a unique ITs pattern across the *Pga* races and did not contain the allele associated with *Pg6*. All four of these diploid accessions warrant additional study to determine if their unique resistance is conferred by novel resistance genes.

Four of the accessions in this study were resistant to DBD but susceptible to all the other races tested. This type of race-specific resistance has not been previously documented within A-genome *Avena* species. This resistance could be conferred by either *Pg2* or *Pg4*, as these genes are effective against race DBD and ineffective against race KBD, or some previously unreported resistance. However, this DBD-only resistance will be ineffective in fields where virulence to these genes is widespread.

Steinberg et al. (2005) identified 35 accessions of the 9978 tested as having high levels of field resistance to oat stem rust. Of these, 33 were susceptible to race NA1 with virulence to *Pg6*, indicating that these accessions may carry *Pg6*. We were able to compare 22 of the accessions in their study using PI/CI accession numbers matching accessions with *Pg6* postulations in the present study and determined that all of them likely contain *Pg6* (Supplementary Table S1). In their study, only two *A. barbata* accessions, CN 23731 and CN 26171, were resistant to NA1 with IT of 0;1, which was more pronounced resistance than the IT of ‘2’ exhibited by the resistant tetraploid accessions in the present study, and may represent another novel source of resistance.

Five *A. vaviloviana* accessions from Oromīya, Ethiopia, had resistant ITs of ‘2’ across the oat stem rust races tested (Table 4). *A. vaviloviana* is an allotetraploid species with an AB-genome that is closely related to *A. barbata* Pott ex Link and *A. abyssinica* Hochst. (Chew et al. 2016; Yan et al. 2016). Intermediate levels of field resistance to oat stem rust were previously reported at low frequency among tested accessions in all three species (Steinberg et al. 2005). Given their similar origin, collection date, and IT, these five *A. vaviloviana* accessions likely contain a single novel source of resistance which warrants further investigation. *A. barbata* and *A. abyssinica* may harbor additional novel alleles, and accessions from these species and other tetraploids should be tested against important oat stem rust races DBD, TJS and TQL to identify additional resistant sources.

All 56 *Pg6*-carrying accessions from the diversity panel were A-genome diploids with A_s_ or A_l_ genomes (Table 1). Maughan et al. (2019) demonstrated that *A. atlantica*, *A. strigosa*, and *A. wiestii* constitute a single species complex differentiated by seed dispersal mechanisms, whereas *A. brevis* could not be genetically differentiated from *A. strigosa*. Fifty-one of the postulated *Pg6*-carrying accessions were in this A_s_ clade and six accessions were in the distantly related *A. longiglumis* clade. The SNP marker most closely associated with the *Pg6* phenotype in the diversity panel matched the *Pg6* phenotype in all but one of the A_s_-genome accessions, but did not align with the *Pg6* phenotypes associated with *A. longiglumis* accessions (Table 3). *A. longiglumis* has a distinct morphology from other A-genome species, is distantly related to other A-genome diploids, and is thought to be the progenitor of all extant *Avena* hexaploids (Yan et al. 2016). It would be interesting to understand whether the resistance in *A*_s_ and *A*_l_ genome diploids is conferred by the same gene or different race-specific genes that have identical resistance patterns across stem rust races. Cloning *Pg6* in *A. strigosa* and mapping the *Pg6*-like resistance in *A. longiglumis* would expand understanding of how resistance arose in A-genome *Avena* accessions and might provide valuable insights into race-specific resistance gene evolution.

Only 11 C-genome accessions were available for testing, and they were susceptible to all four oat stem rust races used in this study (Table 2). A more exhaustive investigation utilizing C-genome accessions from other collections would be required to conclude that oat stem rust resistance is not present in C-genome diploids. Recent genetic studies proposed that speciation between the A- and C-genome diploids occurred between 5.4 and 12.9 million years ago and subsequent tetraploidization and hexaploidization events likely occurred during the Miocene-Pliocene interval in northwest Africa (Chew et al. 2016; Liu et al. 2017; Maughan et al. 2019). If further testing verifies that the *Pg6* phenotype is present in only A-genome diploids, then *Pg6* may have arisen after the A-, C-genome diploid speciation event and was absent in the diploid progenitors of current tetraploid and hexaploid species.

Eight of the *Pg6* carrying accessions showed mixed IT reactions (Table 3). Mixed IT reactions indicate the importance of deriving accessions from a single seed source and retesting the derived line to confirm the phenotype before proceeding with further genetic testing. Mixed accessions can also make it difficult to draw conclusive associations between previously genotyped or sequenced materials and current phenotyping efforts. Care was taken in this study to choose accessions with clear phenotypic responses for SNP development.

CIav 6956, the *Pg6* carrier, showed strong seedling resistance and moderate field resistance to crown rust (T. Gordon, unpublished). Crown rust resistance in another *A. strigosa* accession PI 258731 is remarkably stable and has been introgressed into hexaploid oat (Rines et al. 2018). Another broadly effective source of oat crown rust resistance, *Pc94*, was introduced from the *A. strigosa* accession PI 186606, ‘Saia’ from Rio Grande do Sul, Brazil. The molecular markers that have been developed for the crown rust resistance loci in PI 258731 and *Pc94* are on *A. atlantica* chromosome scaffolds ScoFOjO_350_483 and ScoFOjO_324_449, respectively, whereas *Pg6* resistance was localized to ScoFOjO_1702_2338. These results support a hypothesis that resistance to these rusts is derived from different chromosomal regions, but the relationship between rust resistance loci within *A. strigosa* warrants further investigation.

Kebebe et al. (2020a) mapped the oat stem rust gene *Pg13* between 67.7 and 68.5 cM on hexaploid linkage group Mrg 18. The diagnostic markers reported for *Pg13* in their study were between 491,649,525 and 498,515,330 bp on the diploid chromosome AA2. They also found that the oat crown rust resistance gene *Pc91* co-segregated with *Pg13* on Mrg 18 at the 7C-17A translocation breakpoint. *Pc91* was originally introgressed into *A. sativa* cultivars from the synthetic hexaploid, ‘Amagalon,’ CIav 9364. This line was produced by crossing the tetraploid *A. magna* accession, CIav 8330, with the *A. longiglumis* line, ‘CW 57,’ but it is not documented which species contributed this resistance (Rothman 1984). It is apparent that these three rust resistance genes, *Pg6*, *Pg13*, and *Pc91*, are very close to one another on the A-genome. However, *Pg6* and *Pg13*, show different race specificity (Supplementary Table S1) and the marker most closely associated with *Pg6* in the present study, AA2_483439497, is at least 8 Mbp proximal to the markers closest to *Pg13* and *Pc91*. Additional testing also indicated that Amagalon is susceptible to *Pga* race KBD (T. Gordon, unpublished). A comparative sequencing technique, like the one presented in the current study, could be used to elucidate the relationship between *Pg6*, *Pg13*, and *Pc91*.

Maughan et al. (2019) previously annotated 1,563 RGAs within the *A. atlantica* genome which typically clustered in sub-telomeric regions. In this study, three clusters of SNPs aligned perfectly with the *Pg6* phenotype in the genomic region between 475 and 490 Mbp on AA2 (Fig. 2). The first cluster was composed of 1,138 SNPs, between 478.5 and 479.4 Mbp, the second was composed of 129 SNPs between 482.0 and 482.4 Mbp and the third was composed of 69 SNPs between 483.4 and 483.6 Mbp. Within the first large SNP cluster there was one RGA, a leucine-rich repeat receptor-like protein kinase (LRRK) in a 3 kb section beginning at 478,733,268 bp and annotated as ‘AA012417’ in the *A. atlantica* genome. Most SNPs with perfect association in this region were located slightly downstream from this LRRK gene. However, one SNP located at 478,733,705 bp was within this gene. In contrast, the assay that interrogated this SNP and other SNPs in the first cluster were several cM away from the resistance locus in the RIL population (Table 6).

Another RGA, a 5 kb resistance to Peronospora Parasitica protein 13 (RPP13) between 483,422,214 and 483,427,403 bp and annotated as ‘AA012586’ was located in the third SNP cluster. RPP13 is an NBS-LRR protein which initiates a race-specific hypersensitive response in *Arabidopsis thaliana* when challenged with the obligate biotrophic oomycete pathogen, *Hyaloperonospora arabidopsidis* (Rentel et al. 2008). The interaction between the cloned effector ATR13 and RPP13 elicits a common defense response that was effective against oomycete, viral, and bacterial pathogens (Rentel et al. 2008). Assays used to interrogate SNPs in the region close to the RPP13 analog were predictive of *Pg6*, specifically, marker AA2_483439497 was perfectly aligned with the *Pg6* phenotype in the mapping populations and within the A_s_ genome accessions in the diversity panel (Table 6). This marker was flanked by two SNPs, AA2_483429191 and AA2_483451960, that were slightly less predictive of the *Pg6* phenotype. Oddly, the SNP within the RPP13 gene sequence region AA2_483427147, and the SNP only 2 kb distal to the gene, AA2_483429191, were less predictive of the *Pg6* phenotype than AA2_483439497 which was 12 kb distal indicating a slight rearrangement from the expected gene sequence. Nevertheless, since no other annotated RGA genes were found in this region, these results provide strong support for RPP13 as the candidate *Pg6* resistant gene.

NBS-LRR type genes are effective at controlling biotrophic and hemibiotrophic pathogens, but wide deployment of this type of gene has been problematic in the case of necrotrophic pathogens. Susceptibility to Victoria Blight caused by the necrotrophic fungal pathogen *Bipolaris victoriae* was shown to be conferred by the same NBS-LRR resistance gene that conferred resistance to crown rust caused by the biotrophic fungal pathogen *Pca*, and wide deployment of this type of resistance could induce susceptibility to necrotrophic pathogens (Lorang et al. 2007). Despite the close proximity of the most diagnostic SNPs to an NBS-LRR gene, a causal association has not been made, and further expression, annotation, and gene cloning studies will be required to elucidate a mechanism for *Pg6* resistance.

In conclusion, *Pg6* is a widely effective oat stem rust resistant gene, and SNP markers closely linked with this gene enabled identification of novel sources of oat stem rust resistance from within a diverse collection of *Avena* diploid germplasm. A comparative sequencing technique was used to quickly narrow a genomic region of interest and select a candidate RGA. The utility of the SNPmarker at 483,439,497 bp on AA2 was validated in diverse germplasm and can be used to screen additional germplasm collections and assist with introgression and gene pyramiding of *Pg6*.

## Supplementary Information

Below is the link to the electronic supplementary material.

Supplementary file1 (DOCX 18 kb)

Supplementary file2 (XLSX 40 kb)

Supplementary file3 (XLSX 14 kb)

Supplementary file4 (XLSX 443 kb)

Supplementary file5 (XLSX 125 kb)

Supplementary file6 (XLSX 14 kb)

## Declarations

### Data availability

All data and material used in this study can be accessed. Details are provided in the Materials and Methods Section.

## References

[CR1] Adhikari K, McIntosh R, Oates J (2000). Distribution and temperature sensitivities of genes for stem rust resistance in Australian oat cultivars and selected germplasm. Aust J Agric Res.

[CR2] Blake VC, Woodhouse MR, Lazo GR, Odell SG, Wight CP, Tinker NA, Wang Y, Gu YQ, Birkett CL, Jannink JL (2019) GrainGenes: centralized small grain resources and digital platform for geneticists and breeders. Database 2019 10.1093/database/baz065 PMC6580076 31210272

[CR3] Boshoff W, Visser B, Terefe T, Pretorius Z (2019). Diversity in Puccinia graminis f. sp. avenae and its impact on oat cultivar response in South Africa. Eur J Plant Pathol.

[CR4] Carson ML (2008). Virulence frequencies in oat crown rust in the United States from 2001 through 2005. Plant Dis.

[CR5] Chew P, Meade K, Hayes A, Harjes C, Bao Y, Beattie AD, Puddephat I, Gusmini G, Tanksley SD (2016). A study on the genetic relationships of Avena taxa and the origins of hexaploid oat. Theor Appl Genet.

[CR6] Danecek P, Auton A, Abecasis G, Albers CA, Banks E, DePristo MA, Handsaker RE, Lunter G, Marth GT, Sherry ST (2011). The variant call format and VCFtools. Bioinformatics.

[CR7] FAOSTAT (2020) Production and yield quantities of cereal grains. Food and Agriculture Organization of the United Nations

[CR8] Fardet A (2010). New hypotheses for the health-protective mechanisms of whole-grain cereals: what is beyond fibre?. Nut Res Rev.

[CR9] Federizzi L, Mundstock C, Suttie J, Reynolds S (2004). Fodder oats: an overview for South America. Fodder oats: a world overview plant production and protection series.

[CR10] Fetch TG, Jin Y (2007). Letter code system of nomenclature for Puccinia graminis f. sp. avenae. Plant Dis.

[CR11] Hoffman LA, Livezey J (1987) The US oats industry. US Department of Agriculture, Economic Research Service

[CR12] Kebede AZ, Admassu-Yimer B, Bekele WA, Gordon T, Bonman JM, Babiker E, Jin Y, Gale S, Wight CP, Tinker NA (2020). Mapping of the stem rust resistance gene Pg13 in cultivated oat. Theor Appl Genet.

[CR13] Kebede AZ, Bekele WA, Mitchell Fetch JW, Beattie AD, Chao S, Tinker NA, Fetch TG, McCartney CA (2020). Localization of the stem rust resistance gene *Pg2* to linkage group Mrg20 in cultivated oat (Avena sativa). Phytopathology.

[CR14] Kosgey ZC, Edae EA, Dill-Macky R, Jin Y, Bulbula WD, Gemechu A, Macharia G, Bhavani S, Randhawa MS, Rouse MN (2021). Mapping and validation of stem rust resistance loci in spring wheat line CI 14275. Front Plant Sci.

[CR15] Li T, Cao Y, Wu X, Chen S, Wang H, Li K, Shen L (2015). First report on race and virulence characterization of Puccinia graminis f. sp. avenae and resistance of oat cultivars in China. Eur J Plant Pathol.

[CR16] Liu Q, Lin L, Zhou X, Peterson PM, Wen J (2017). Unraveling the evolutionary dynamics of ancient and recent polyploidization events in Avena (Poaceae). Sci Rep.

[CR17] Lorang JM, Sweat TA, Wolpert TJ (2007). Plant disease susceptibility conferred by a “resistance” gene. Proc Nat Acad Sci.

[CR18] Martens J, Roelfs A, Bushnell W (1985). Oat stem rust. The cereal rusts.

[CR19] Maughan PJ, Lee R, Walstead R, Vickerstaff RJ, Fogarty MC, Brouwer CR, Reid RR, Jay JJ, Bekele WA, Jackson EW (2019). Genomic insights from the first chromosome-scale assemblies of oat (Avena spp.) diploid species. BMC Biol.

[CR20] O'Donoughue LS, Chong J, Wight CP, Fedak G, Molnar SJ (1996). Localization of stem rust resistance genes and associated molecular markers in cultivated oat. Phytopathology.

[CR21] PepsiCo (2020) Avena sativa – OT3098 v1

[CR22] Pomeranz Y, Robbins GS, Briggle LW (1971). Amino acid composition of oat groats. J Agr Food Chem.

[CR23] Rentel MC, Leonelli L, Dahlbeck D, Zhao B, Staskawicz BJ (2008). Recognition of the Hyaloperonospora parasitica effector ATR13 triggers resistance against oomycete, bacterial, and viral pathogens. Proc Nat Acad Sci.

[CR24] Rimmer A, Phan H, Mathieson I, Iqbal Z, Twigg SR, Wilkie AO, McVean G, Lunter G (2014). Integrating mapping-, assembly-and haplotype-based approaches for calling variants in clinical sequencing applications. Nat Genet.

[CR25] Rines HW, Miller ME, Carson M, Chao S, Tiede T, Wiersma J, Kianian SF (2018). Identification, introgression, and molecular marker genetic analysis and selection of a highly effective novel oat crown rust resistance from diploid oat, Avena strigosa. Theor Appl Genet.

[CR26] Roelfs AP, Long DL (1980). Analysis of recent oat stem rust epidemics. Phytopathology.

[CR27] Rothman P (1984). Registration of four stem rust and crown rust resistant oat germplasm lines. Crop Sci.

[CR28] Rouse MN, Talbert LE, Singh D, Sherman JD (2014). Complementary epistasis involving Sr12 explains adult plant resistance to stem rust in thatcher wheat (Triticum aestivum L.). Theor Appl Genet.

[CR29] Sika KC, Kefela T, Adoukonou-Sagbadja H, Ahoton L, Saidou A, Baba-Moussa L, Baptiste LJ, Kotconi SO, Gachomo EW (2015). A simple and efficient genomic DNA extraction protocol for large scale genetic analyses of plant biological systems. Plant Gene.

[CR30] Stakman EC, Levine M, Bailey D (1923). Biologic forms of Puccinia graminis on varieties of Avena spp. J Agric Res.

[CR31] Stakman EC, Steward DM, Loegering WQ (1962) Identification of physiologic races of Puccinia graminis var. *tritici*. USDA Agric Res Serv E-617, Washington, DC

[CR32] Steinberg JG, Fetch JM, Fetch TG (2005). Evaluation of Avena spp. accessions for resistance to oat stem rust. Plant Dis.

[CR33] Torkamaneh D, Laroche J, Tardivel A, O'Donoughue L, Cober E, Rajcan I, Belzile F (2018). Comprehensive description of genomewide nucleotide and structural variation in short-season soya bean. Plant Biotechnol J.

[CR34] Van Niekerk B, Pretorius Z, Boshoff W (2001). Potential yield losses caused by barley leaf rust and oat leaf and stem rust to South African barley and oat cultivars. S Afri J Plant Soil.

[CR35] Winkler LR, Murphy KM, Hermes JC (2018). Three hulless oat varieties show economic potential as organic layer feed grain. Renew Agr Food Syst.

[CR36] Yan H, Bekele WA, Wight CP, Peng Y, Langdon T, Latta RG, Fu Y-B, Diederichsen A, Howarth CJ, Jellen EN, Boyle B, Wei Y, Tinker NA (2016). High-density marker profiling confirms ancestral genomes of *Avena* species and identifies D-genome chromosomes of hexaploid oat. Theor Appl Genet.

