# Supplementary material for: Comparative sequencing and SNP marker validation for oat stem rust resistance gene Pg6 in a diverse collection of Avena accessions
Source: Theor Appl Genet. 2022 Feb 3;135(4):1307–18. doi: 10.1007/s00122-022-04032-z (PMC9033690; doi:10.1007/s00122-022-04032-z)
Supplement: Supplementary file 1 — Supplementary file1 (DOCX 18 kb) [file 122_2022_4032_MOESM1_ESM.docx]

**Supplementary Fig. S1** SNP allele accuracy based on *Pg6* phenotype postulation tested in two bi-parental mapping populations and a diverse panel of 127 *A. strigosa* accessions; 573582/*Pg6* F_5:6_ RILs represented by blue triangles; 2524/*Pg6* F_2:3_ families represented with yellow xs; and the *A. strigosa* panel represented by purple squares
